# Multi-omics analysis reveals overactive inflammation and dysregulated metabolism in severe community-acquired pneumonia patients

**DOI:** 10.1186/s12931-024-02669-6

**Published:** 2024-01-19

**Authors:** Jieqiong Li, Yawen Wang, Weichao Zhao, Tingyu Yang, Qianyu Zhang, Huqin Yang, Xuyan Li, Zhaohui Tong

**Affiliations:** 1 Medical Research Center, Beijing Institute of Respiratory Medicine and Beijing Chao-Yang Hospital, Capital Medical University, 8 Workers Stadium South Road, Chaoyang District, Beijing, China; 2 Department of Respiratory and Critical Care Medicine, Beijing Institute of Respiratory Medicine, Beijing Chao-Yang Hospital, Capital Medical University, 8 Workers Stadium South Road, Chaoyang District, Beijing, China; 3 Department of Respiratory and Critical Care Medicine, Tianjin Chest Hospital, Tianjin, China; 4 Department of Respiratory Medicine, Strategic Support Force Medical Center, Beijing, China

**Keywords:** Severe community-acquired pneumonia, Proteomics, Metabolomics, Biomarker, Pathogenesis

## Abstract

**Background:**

Severe community-acquired pneumonia (S-CAP) is a public health threat, making it essential to identify novel biomarkers and investigate the underlying mechanisms of disease severity.

**Methods:**

Here, we profiled host responses to S-CAP through proteomics analysis of plasma samples from a cohort of S-CAP patients, non-severe (NS)-CAP patients, diseases controls (DCs), and healthy controls (HCs). Then, typical differentially expressed proteins were then validated by ELISA in an independent cohort. Metabolomics analysis was further performed on both the cohort 1 and cohort 2. Then, the proteomic and metabolomic signatures were compared between the adult and child cohorts to explore the characteristics of severe pneumonia patients.

**Results:**

There were clear differences between CAP patients and controls, as well as substantial differences between the S-CAP and NS-CAP. Pathway analysis of changes revealed excessive inflammation, suppressed immunity, and lipid metabolic disorders in S-CAP cases. Interestingly, comparing these signatures between the adult and child cohorts confirmed that overactive inflammation and dysregulated lipid metabolism were common features of S-CAP patients, independent of age. The change proportion of glycerophospholipids, glycerolipids, and sphingolipids were obviously different in the adult and child S-CAP cases.

**Conclusion:**

The plasma multi-omics profiling revealed that excessive inflammation, suppressed humoral immunity, and disordered metabolism are involved in S-CAP pathogenesis.

**Supplementary Information:**

The online version contains supplementary material available at 10.1186/s12931-024-02669-6.

## Background

Community-acquired pneumonia (CAP) is a major health burden that causes great morbidity and mortality worldwide [1]. A previous study revealed that urban China has a high prevalence of CAP, with an overall incidence of approximately 7.13 per 1000 person-years, meriting widespread attention [2]. Approximately 8–20% of hospitalised CAP patients, especially older patients and those with comorbidities, progress to severe disease (S-CAP), and many require admission to the intensive care unit (ICU). Moreover, as many as 25% of these severe patients require advanced interventions, such as intubation, to reduce the mortality rate [3].

Omics analysis has been shown to efficiently identify drug targets and biomarkers to predict the severity and progression of diseases [4]. As a less invasive sample type, plasma and serum store large numbers of small molecules, and their abundance can provide valuable information for biomarker selection and pathogenesis analysis [5]. Our previous studies have focused on plasma or serum and integrating multi-omics analysis of diseases, including respiratory syncytial virus pneumonia [6] and *Mycoplasma pneumoniae* pneumonia [7]. For example, our previous study identified a diagnostic panel to accurately distinguish respiratory syncytial virus infection from controls [6], while in another study, metabolomics was combined with a random forest-based classification model to identify potential biomarkers for the diagnosis of *Mycoplasma pneumoniae* pneumonia [7].

To date, only a few reported studies have aimed to identify specific proteins or metabolites associated with the severity of CAP. One study reported that plasma metabolites can be used to assess disease severity and predict the 90-day mortality prognosis of patients with bacterial pneumonia [8]. Another study suggested that a metabolite panel combining lactate, sphinganine, and dehydroepiandrosterone sulfate could be used for severity assessment of CAP, with a better ability than that of the CURB-65 or PSI and as well as that of APACHE II scores [9]. Our recent study identified a diagnostic panel to distinguish patients with paediatric S-CAP from those with NS-CAP and healthy donors [10]. However, there are significant differences in the immune system and metabolic responses between adults and children, especially during disease progression. The characteristics of S-CAP in adults, as well as the commonalities and differences of S-CAP signatures between children and adults, need to be further investigated. Moreover, our previous work lacked a disease control (DC), as distinguish between CAP and other respiratory diseases is urgently needed. Thus, an integrated analysis of the proteomic and metabolomic data was still essential for the understanding of adult S-CAP.

Here, we used proteomic and metabolomic technologies to analyse the proteome and metabolome in plasma samples from S-CAP patients, NS-CAP patients, healthy controls (HCs), and DCs. The goals of this study were: (1) to elucidate the molecular changes associated with S-CAP and investigate the underlying pathological mechanisms; and (2) to compare the commonalities and differences of host responses to S-CAP between adult and child cohorts. Our trans-omics insights will contribute to providing a comprehensive understanding of the underlying pathogenesis of S-CAP.

## Materials and methods

### Patients

All 161 subjects including 31 S-CAP, 43 NS-CAP and 42 DC patients, as well as 45 HCs were enrolled from Beijing Chao-Yang Hospital during April 2022 to January 2023 (Additional file 1: Table S1). Most CAP samples were collected at the start (Day1 or Day2) of hospitalization (Additional file 2: Fig. S1). This study was reviewed and approved by the Ethics Committee of Beijing Chao-Yang Hospital (2021-Ke-501). Written informed consent for sample collection and analysis was provided by all participants in the current study in accordance with the Declaration of Helsinki.

Patients with CAP were diagnosed according to the following criteria: (1) community onset; (2) one of the following clinical presentations of pneumonia: fever; new-onset cough or expectoration or worsening of existing respiratory diseases, with or without respiratory distress, purulent sputum, chest pain, or haemoptysis; physical examination revealing lung consolidation or wet rales; peripheral blood leukocytes > 10 × 10^9^/L or < 4 × 10^9^/L, with or without left shift; (3) chest X-ray or computed tomography suggesting new abnormalities including patchy exudations, lobar or segmental consolidation, ground-glass opacities, or interstitial changes, with or without pleural effusion. Patients were diagnosed with CAP if they satisfied all three criteria simultaneously and the criteria were not attributed to other respiratory diseases, including tuberculosis, lung tumour, non‐infectious interstitial lung disease, pulmonary oedema, atelectasis, pulmonary embolism, pulmonary eosinophilia, or pulmonary vasculitis [11].

Disease severity was defined as severe or non-severe. Briefly, patients who met one major criterion or more than three minor criteria were diagnosed with severe infection. Accordingly, major criteria included: (1) tracheal intubation required for invasive mechanical ventilation and (2) septic shock requiring vasoactive agent administration following active fluid resuscitation. Minor criteria were as follows: respiratory rate equal to or greater than 30 times per minute; oxygenation index equal to or less than 250 mmHg; multi-lobar infiltrates; blood urea nitrogen equal to or greater than 7.14 mmol/L; consciousness disorder or disorientation; and systolic blood pressure less than 90 mmHg with a need for active fluid resuscitation.

Furthermore, patients enrolled as DCs were diagnosed with non-infectious respiratory diseases and the diagnosis of pneumonia was excluded. Meanwhile, HCs without respiratory diseases were recruited from the physical examination centre of Beijing Chao-Yang Hospital.

### Clinical information

Patients’ clinical symptoms and laboratory data were collected for further analysis using the hospital’s electronic medical records. Recorded laboratory results included procalcitonin, peripheral white blood cell count, neutrophil proportion, lymphocyte proportion, monocyte proportion, prothrombin time, activated partial thrombin time, fibrinogen, D-dimer, thrombin time, and other informative indexes (Additional file 3: Table S2).

### Proteomics analysis

We performed a proteomics analysis on plasma samples from the 40 individuals of the cohort 1 [12]. In accordance with the manufacturer’s instructions, all samples were lysed in lysis buffer at 25 °C for 30 min and then reduced and alkylated using tris phosphine (Pierce, USA) and iodoacetamide (Sigma-Aldrich, USA), respectively. Proteins were digested overnight with a mixture of mass spectrometry-grade trypsin gold (Promega, USA). We further conducted liquid chromatography-tandem mass spectrometry (LC-MS/MS) via an Orbitrap Fusion Lumos in the data-dependent acquisition mode, which was coupled with the UltiMate 3000 HPLC system (Thermo Fisher Scientific, Waltham, MA USA). Peptides were quantified according to the peak area of MS1 intensity, and then the intensity of unique and razor peptides was used to calculate the peptide intensity. For proteomics, the resultant mass spectrometry data were analyzed using Maxquant (Version 2.1.4.0) and the protein search database was the Homo sapiens FASTA database downloaded from UniprotKB (UP000005640_9606_one_gene_one_protein.fasta). The Maxquant LFQ intensity table were loaded into the Perseus_2.0.7.0; R 4.2. Then, quantitative values were converted according to log2. At least one group sample containing more than 5 quantitative values was retained. After the missing values were filled by normal distribution method, subsequent inter-group comparative analysis was conducted.

### Enzyme-linked immunosorbent assay (ELISA)

ELISAs were used to detect the levels of selected proteins in plasma samples from the validation cohort patients. Serum amyloid A-1 protein (SAA1), SAA2, pulmonary surfactant-associated protein B (SFTPB), C-reactive protein (CRP), inter-alpha-trypsin inhibitor heavy chain H1 (ITIH1), transketolase (TKT), phosphatidylcholine-sterol acyltransferase (LCAT), lipopolysaccharide (LPS)-binding protein (LBP), cholesteryl ester transfer protein (CETP), glucose-6-phosphate isomerase (GPI), and L-lactate dehydrogenase A (LDHA) levels were examined using ELISA kits.

### Metabolomics analysis

Metabolomics analysis was conducted on these plasma samples as previously described [12]. Ultra High Performance Liquid Chromatography-Mass Spectrum (UHPLC-MS/MS) analyses were conducted in positive or negative polarity mode and performed on a ACQUITY UPLC I-Class system (Thermo Fisher Scientific, Waltham, MS, USA) coupled with an Orbitrap Q-Exactive mass spectrometer equipped with a heated electrospray ionization (ESI) source (Thermo Fisher Scientific, Waltham, MS, USA). For metabolomics, metabolite identifications were supported by the Human Metabolome Database (HMDB), Lipidmaps(v2.3) and METLIN. We set quality control (QC) for normalization, and pooled QC samples were prepared by mixing equal amounts of plasma from all samples. The auto data scaling method was mean-centered and divided by the standard deviation of each variable. The pretreatment of the QC samples was performed in parallel with other samples. The QC samples were evenly inserted between each set of runs to monitor the stability of the large-scale analysis.

### Statistical analysis

Fold changes in proteins and metabolites were calculated using the mean relative abundance across patients in each pair of comparison groups. The differentially expressed proteins (DEPs) or differentially expressed metabolites (DEMs) were defined as *P*-values < 0.05 and fold changes ≥ 1.5 or < 0.67. Statistical significance in multigroup analyses were calculated by one-way analysis of variance (ANOVA) and Tukey’s honestly significant difference (HSD) test. MetaboAnalyst 5.0 was then used to perform partial least squares-discriminate analysis (PLS-DA). Heatmaps were generated using the “pheatmap” package (v1.0.12), while bubble plots, box plots, and bar plots were implemented in the “ggplot2” package (v3.3.6) in R language. A Web accessible resource was used to determine the over representation of gene ontology (GO) categories. Signaling pathway analysis was performed by the Kyoto Encyclopedia of Genes and Genome (KEGG) database. Connected networks were built using Cytoscape (v.3.2.1).

## Results

### Sample cohort and study design

**Fig. 1:**
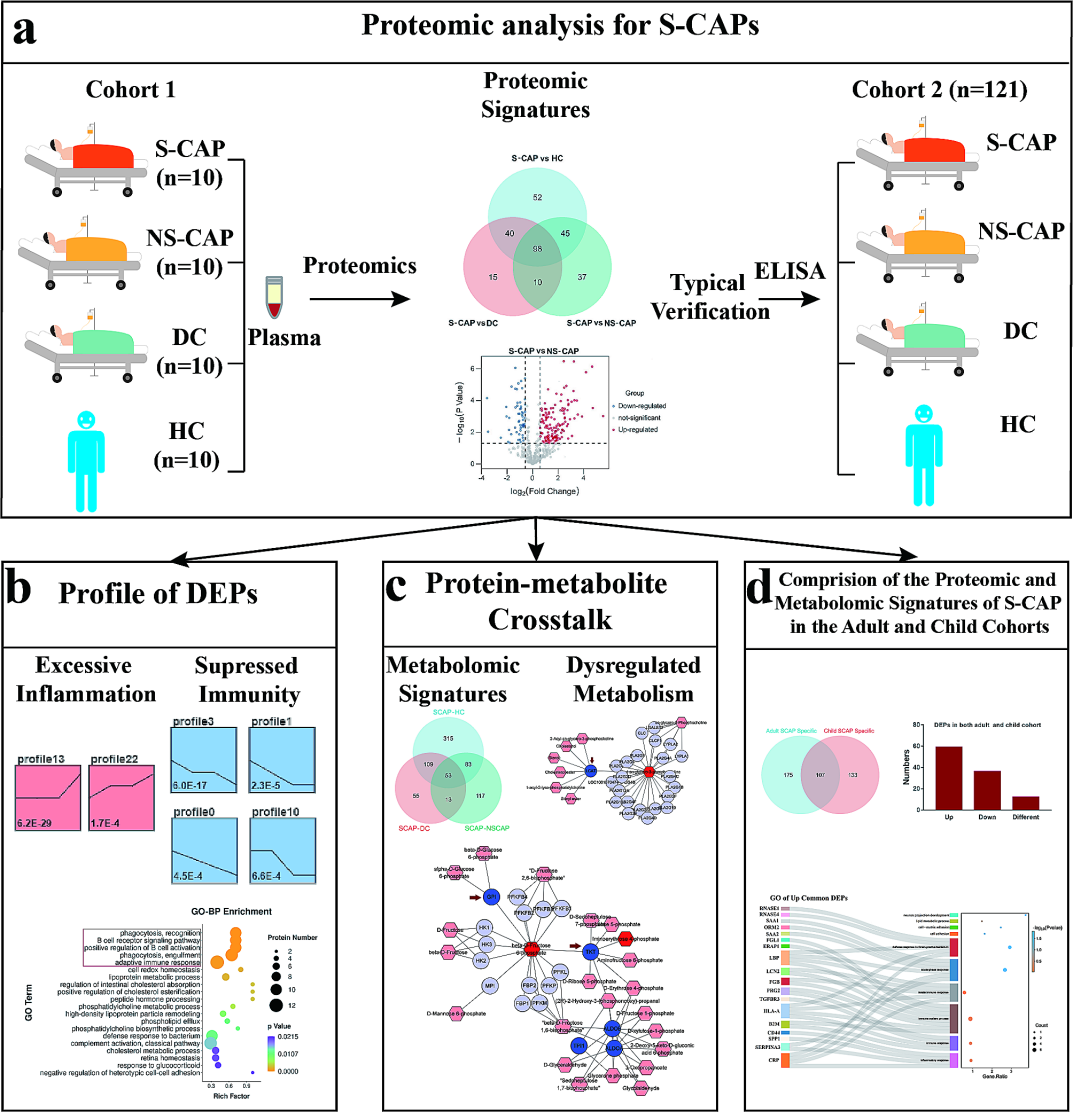
Study overview. (**a**) Study design. Plasma samples from 40 individuals (10 severe community-acquired pneumonia (S-CAP) cases, 10 healthy controls (HCs), 10 disease controls (DCs), and 10 non-severe (NS)-CAP cases of the cohort 1 were collected for proteomics analysis. Eight typical differentially expressed proteins (DEPs) were verified in the cohort 2 (*n* = 121) using enzyme-linked immunosorbent assays (ELISAs). (**b**) Proteomic features of the samples were examined using clustering and enrichment analyses. (**c**) Metabolomics analysis was performed on plasma samples from all 161 subjects. The combined DEPs and differentially expressed metabolites (DEMs) were used for a protein-metabolite integrated analysis. (**d**) Multi-omics data from adult S-CAP patients were compared with a published pediatric dataset to explore the common features of S-CAP

To explore potential biomarkers and investigate the molecular features of S-CAP, we conducted proteomics analysis on plasma samples derived from cohort 1 that included 10 S-CAP, 10 NS-CAP, 10 DC, and 10 HC individuals (Fig. 1a). From these proteomics data, typical DEPs were verified in an independent validation cohort with ELISAs.

Next, we conducted a functional analysis of the DEPs to gain insights into the molecular signatures of S-CAP. Intriguingly, excessive inflammation, suppressed immunity, and disturbed metabolism were observed in S-CAP samples (Fig. 1b). Then, protein-metabolite crosstalk analysis was conducted to understand the specific mechanisms associated with S-CAP (Fig. 1c). Moreover, a comparison of the S-CAP proteomic and metabolomic signatures in the adult and child cohorts was applied to reveal the common features of S-CAP (Fig. 1d).

### Proteomic analysis for S-CAP

#### Proteomic changes in S-CAP samples

**Fig. 2:**
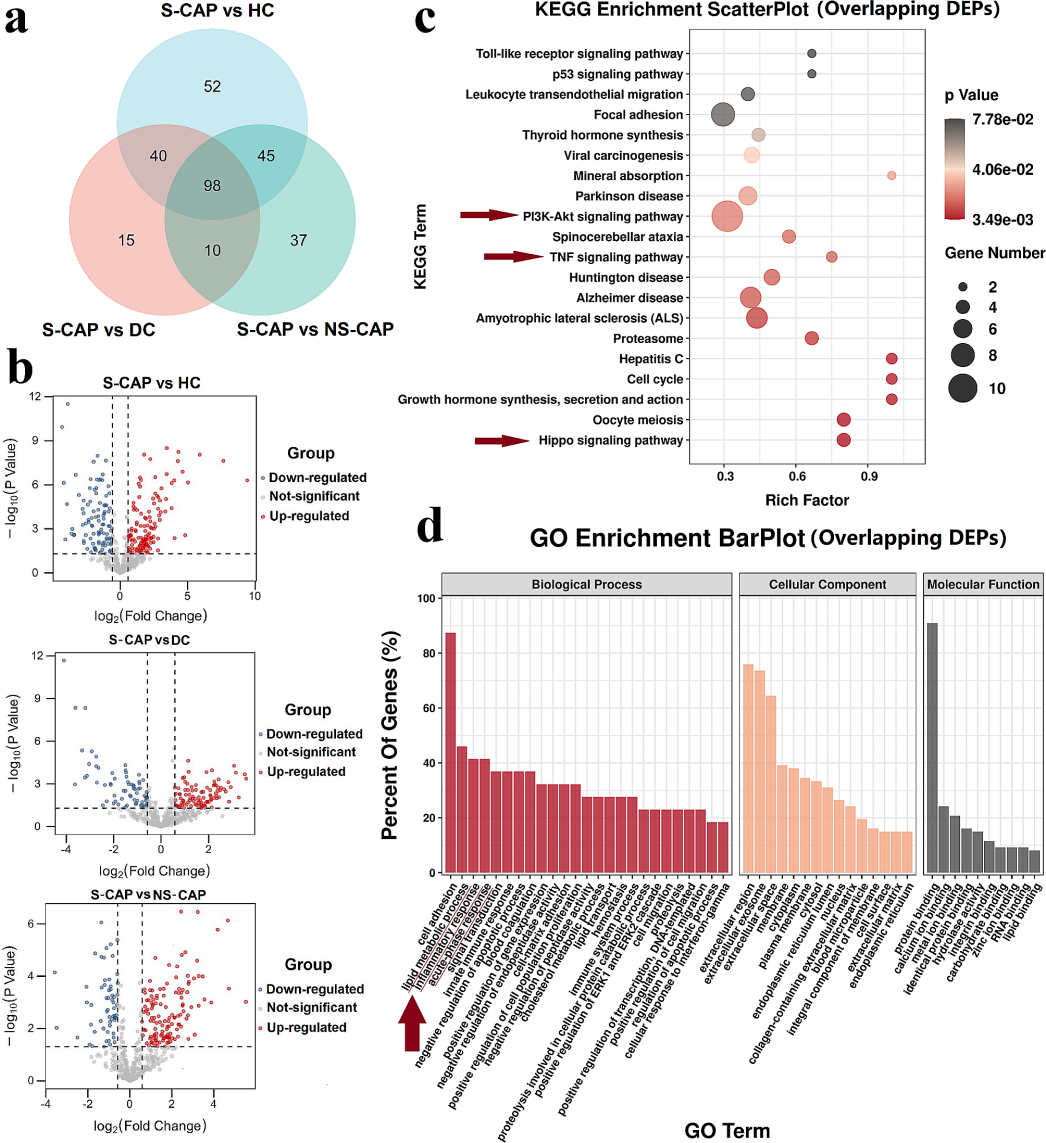
Identification of differentially expressed proteins (DEPs) in severe community-acquired pneumonia (S-CAP) patients from the cohort 1. **a**) A Venn diagram of the number of DEPs. **b**) Volcano plots comparing protein expression in S-CAP vs. healthy controls (HCs), disease controls (DCs), and non-severe (NS)-CAP, respectively. **c**) Kyoto Encyclopedia of Genes and Genomes (KEGG) enrichment analysis of these overlapping DEPs. The top 20 KEGG terms are shown. The arrows highlight the inflammation-related signalling pathways. **d**) Gene ontology (GO) enrichment analysis of these overlapping DEPs. The arrow and red lines highlight the metabolic, inflammatory, and immune processes

From the LC-MS/MS analysis, a total of 1,194 proteins (Additional file 2: Fig. S2a) and 9,774 peptides (Additional file 2: Fig. S2b) were quantified. PLS-DA was conducted to ensure the reliability of these results (Additional file 2: Fig. S3). A Venn diagram (Fig. 2a) and volcano plots (Fig. 2b) were used to display the DEPs. Accordingly, we observed 235, 163, and 190 DEPs in the S-CAP group compared with HC, DC and NS-CAP group, respectively (Additional file 4: Table S3.1). Among these, 98 overlapping DEPs were used for further analysis (Fig. 2a). Inflammation related proteins such as SAA1, SAA2 and CRP were top DEPs in S-CAP pathophysiological processes. Notably, KEGG analysis indicated that severe pneumonia had a significant impact on inflammation-related pathways, including the toll-like receptor (TLR), phosphatidylinositol 3-kinase (PI3K)/serine-threonine kinase (Akt), and other pathways (Fig. 2c, red arrows). Consistently, the GO terms were highly enriched in lipid metabolic process, inflammatory response, acute-phase response, and innate immune response (Fig. 2d, red lines). These results suggested that excessive inflammation, an altered immune status, and a metabolic disorder possibly participate in S-CAP pathogenesis.

#### Verification of typical DEPs

**Fig. 3:**
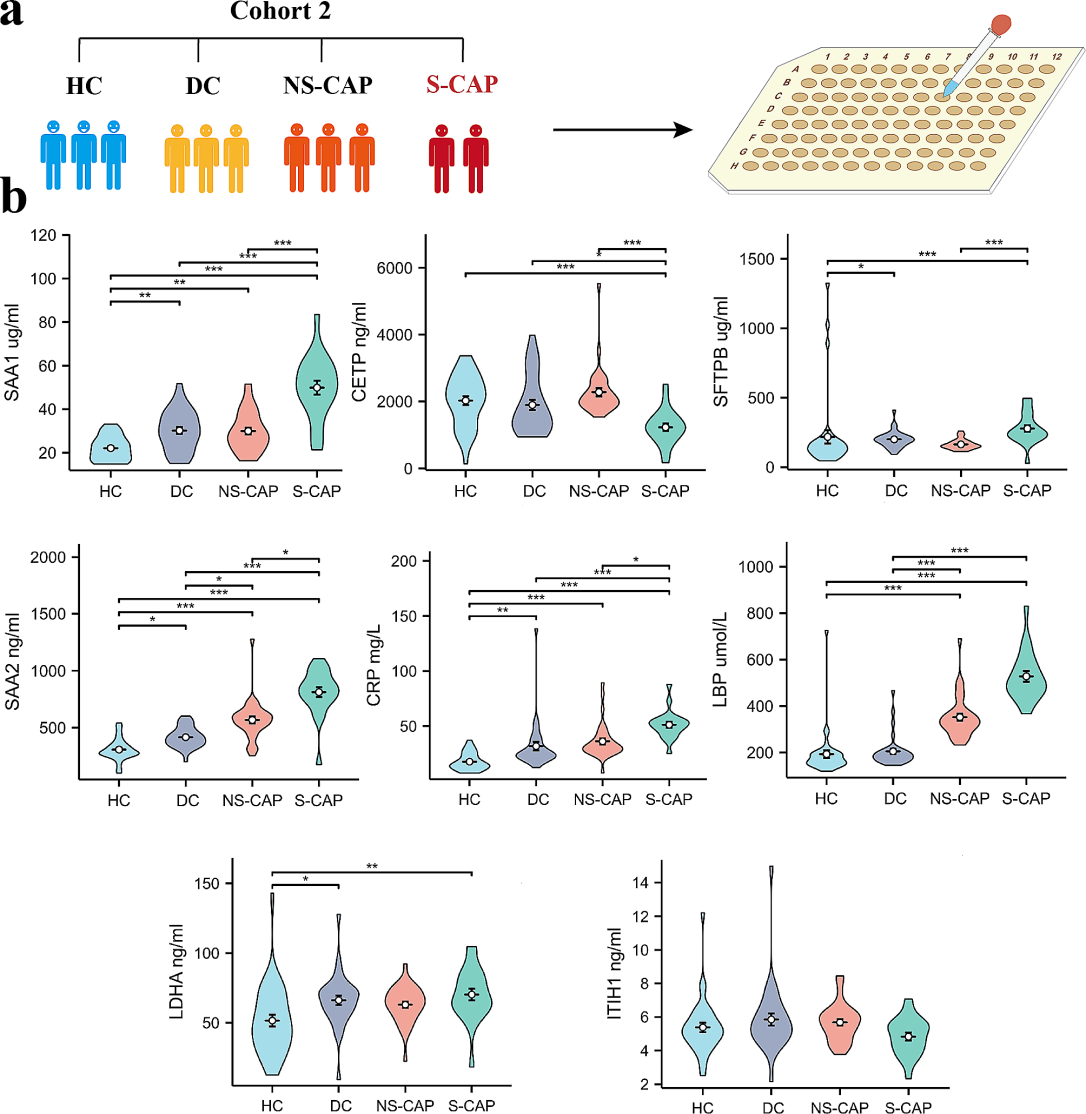
Verification of DEPs in Cohort 2. **a**) Schematic plot. Plasma samples from an independent validation cohort (*n* = 121) were collected and tested using enzyme-linked immunosorbent assays (ELISAs). (**b**) ELISA results of another selected eight DEPs in the cohort 2. Comparisons between two groups were made using Student’s t tests. *P < 0.05, **P < 0.01, and ***P < 0.001

To verify the reliability of proteomics, eight DEPs (SAA1, SAA2, SFTPB, CRP, LBP, CETP, ITIH1, and LDHA) were selected for verification by ELISA (Fig. 3a). The screening criteria for the DEPs were as follows: (1) high fold change; (2) associated with inflammation, immune response, or metabolic disorder.

As shown in Fig. 3b, the results of these selected proteins showed almost consistent trends with the proteomics data. Compared to HC, the levels of inflammation related proteins (SAA1, SAA2, CRP and LBP) were increased in CAPs. Our results confirmed the accuracy of our proteomics data.

### Profile of DEPs

#### Excessive inflammatory response in S-CAP cases

**Fig. 4:**
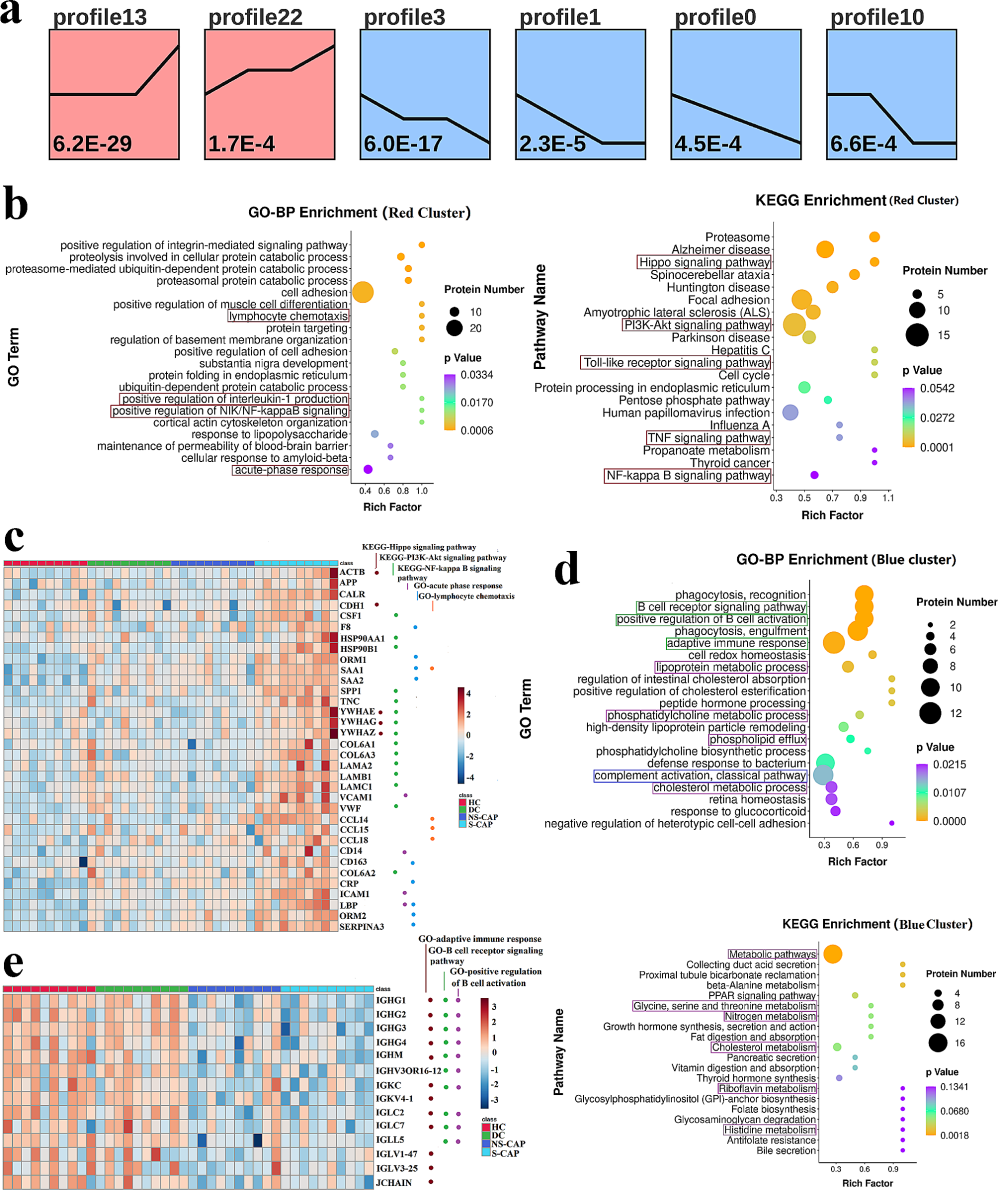
Proteomic signatures of severe community-acquired pneumonia (S-CAP). (**a**) Hierarchical clustering shows seven differential expression patterns across four groups. The red profiles indicate an increasing trend and the blue profiles indicate a decreasing trend. (**b**) Gene ontology (GO) and Kyoto Encyclopedia of Genes and Genomes (KEGG) terms enriched in the red cluster. The top 20 GO terms are shown. The red boxes highlight the inflammation-related pathways. (**c**) Heatmap depicting the levels of differentially expressed proteins (DEPs) related to the Hippo, PI3K-Akt, NF-kappa B, and other signalling pathways in S-CAP cases, healthy controls (HCs), disease controls (DCs), and non-severe (NS)-CAP cases. (**d**) GO and KEGG terms enriched in the blue cluster. The top 20 GO terms are shown. (**e**) Heatmap depicting the levels of DEPs related to the adaptive immune response, B cell receptor signalling pathway, and positive regulation of B cell activation in S-CAP cases, HCs, DCs, and NS-CAP cases

From the proteomics data, we identified two expression patterns: one with a gradually increasing trend (red, profiles 13 and 22) and one with a gradually decreasing trend (blue, profiles 3, 1, 0, and 10) (Fig. 4a). GO and KEGG analyses of the DEPs among the increasing trend showed that they are related to a series of classical inflammatory pathways, such as Hippo, PI3K/Akt, and other signalling pathways (Fig. 4b, red box). For example, a growing number of studies have shown that the Hippo signalling pathway is implicated in pneumonia and acute lung injury [13]. Here, proteins in the Hippo signalling pathway, including ACTB, YWHAE, YWHAG, and YWHAZ, were clearly increased in S-CAP cases (Fig. 4c), indicating the possible presence of acute lung injury in these patients. Moreover, elevated levels of acute phase proteins, including alpha-1-acid glycoprotein (ORM1), ORM2, SAA1, SAA2, CRP, and LBP, were observed in S-CAP (Fig. 4c). Some of these proteins have been reported to be increased in pneumonia [14] and COVID-19 [15] cases. Collectively, upregulation of these proteins potentially results in overactivation of inflammation, as well as tissue damage, during S-CAP pathogenesis.

#### Suppressed humoral immunity in S-CAP cases

We next investigated the functions of the DEPs in the decreasing clusters. Intriguingly, these proteins were mainly enriched in adaptive immunity, especially humoral immunity, phagocytosis, and complement activation, indicating that critically ill pneumonia patients failed to launch a robust B cell-mediated immune response (Fig. 4d, green box). As shown in Fig. 4e, a series of immunoglobulins, such as immunoglobulin heavy constant gamma 1 (IGHG1), IGHG2, and immunoglobulin heavy constant mu (IGHM), were clearly decreased in S-CAP samples. Additionally, a dysregulated complement response was also observed in S-CAP cases (Fig. 4d, blue box). For example, we observed that complement C1q was significantly decreased in S-CAP patients, which is consistent with results from one study that examined COVID-19 patients [16]. Taken together, suppressed immunity was observed in S-CAP cases, suggesting that immunocompromised patients are more likely to develop to severe pneumonia.

### Integrated protein-metabolite crosstalk in S-CAP

#### Metabolomic alterations in S-CAP

In addition to an excessive inflammatory response and suppressed immunity, GO enrichment analysis showed that the predominant terms were associated with metabolism-related pathways, including “lipoprotein metabolic process”, “phosphatidylcholine metabolic process”, “phospholipid efflux”, and “cholesterol metabolic process” (Fig. 4d, purple box). Furthermore, KEGG analysis also revealed that infection had significant impacts on metabolic pathways, glycine, serine, and threonine metabolism, and other pathways (Fig. 4d, purple box).

**Fig. 5:**
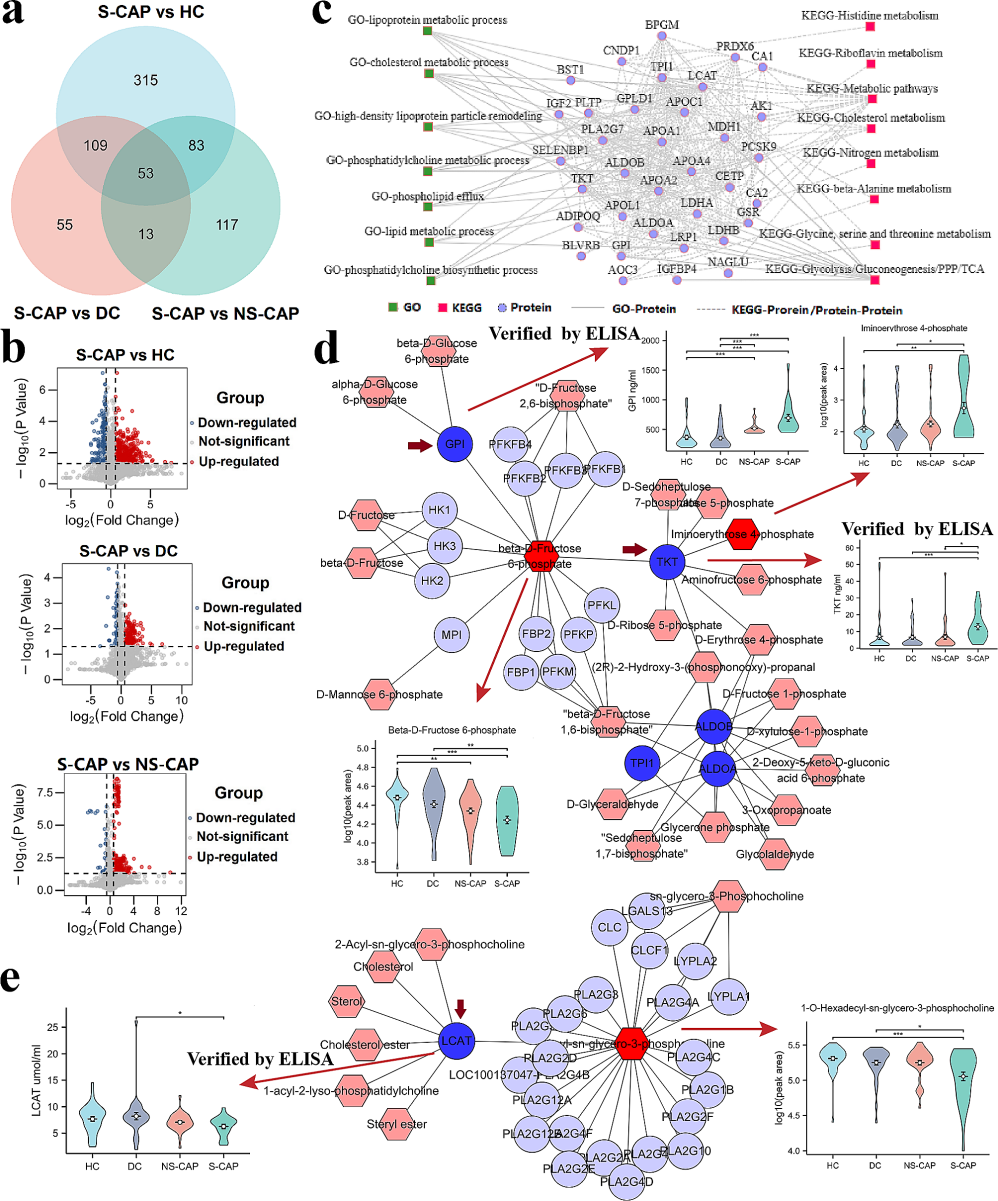
Metabolic signatures and protein-metabolite crosstalk analysis in severe community-acquired pneumonia (S-CAP). (**a**) Venn diagram of the number of differentially expressed proteins (DEPs). (**b**) Volcano plots comparing protein expression in S-CAP vs. healthy controls (HCs), disease controls (DCs), and non-severe (NS)-CAP, respectively. (**c**) Interaction diagram showing the metabolism-related DEPs using Cytoscape. Solid lines indicate associations between proteins in gene ontology (GO) pathways. Dotted lines indicate associations between proteins in Kyoto Encyclopedia of Genes and Genomes (KEGG) pathways. **d**–**e**) Network diagram showing protein-metabolite correlations and boxplots showing enzyme-linked immunosorbent assay (ELISA) results of hub proteins tested in all 161 individuals. Blue circles represent the altered proteins and red rhombuses represent the altered metabolites. Comparisons between two groups were made using Student’s t tests. *P < 0.05, **P < 0.01, and ***P < 0.001

To identify the specific metabolic changes associated with disease progression, comprehensive untargeted metabolomic profiling was conducted in plasma samples from all 161 subjects (including the cohort 1 and cohort 2). After data processing and annotation, a total of 3,345 metabolites were identified. PLS-DA was used to visualize the separation of S-CAP from the other groups (Additional file 2: Fig. S4). A Venn diagram (Fig. 5a) and volcano plots (Fig. 5b) were used to display the DEMs. Accordingly, a total of 745 DEMs were identified in S-CAP cases compared with HCs, DCs, and NS-CAP cases. These DEMs were concentrated in glucose metabolism and lipid metabolism (Additional file 4: Table S3.2). These results are consistent with our findings that the DEPs in the S-CAP cases were significantly dysregulated in metabolic pathways. Among them, (4R,6 S)-6-[(E)-2-[2-(4-Fluoro-3-methylphenyl)-4,6-dimethylphenyl]ethenyl]-4-hydroxyoxan-2-one, Doxofylline and Armillane were the obviously changed in S-CAPs compared to controls.

#### Dysregulated metabolic function in S-CAP

To further understand the interactions of the identified DEPs and DEMs, we firstly categorized the metabolic pathway-related DEPs using the string and cytocape. Among the 15 resulting functional modules, the results indicated that these proteins were mainly enriched in lipid metabolism (lipoprotein metabolic process, lipid metabolic process) and glycometabolic process (glycolysis/gluconeogenesis/pentose phosphate pathway, Fig. 5c). The specialized function of metabolism, comprised of many proteins, was highly enriched for “lipid metabolic process” and “glycometabolic process”. Notably, the proteins belonging to these modules were also connected with each other. Additionally, levels of TKT and GPI, which were associated with glycometabolic process, were significantly increased in S-CAP samples. On the contrary, the levels of most proteins related to lipid metabolic process, including LCAT, apolipoprotein A 1 (APOA1), APOA2, APOA4, apolipoprotein C1 (APOC1), and apolipoprotein L1 (APOL1), were markedly decreased. This further suggested that activation of glycolysis and lactate production, as well as suppression of lipid metabolism, are involved in S-CAP pathogenesis.

Moreover, these metabolism-related proteins and DEMs were selected for crosstalk analysis. As shown in the network diagram, hub proteins, including GPI, TKT (Fig. 5d), and LCAT (Fig. 5e), were centrally located. We then performed ELISAs to verify the levels of these hub proteins in the cohort 2, with the results being consistent with our earlier proteomics data. GPI and TKT levels were increased (Fig. 5d), while LCAT levels were decreased (Fig. 5e), in CAP samples compared with controls. Additionally, the levels of iminoerythrose 4-phosphate and beta-D-fructose 6-phosphate, which are related to glycolysis, were also changed in S-CAP cases (Fig. 5d). Levels of 1-O-Hexadecyl-sn-glycero-3-phosphocholine, which is associated with LCAT, were also significantly decreased in S-CAP samples (Fig. 5e). Collectively, our data suggest that activated glycolysis and lactate production, as well as suppressed lipid metabolism, are involved in S-CAP pathogenesis.

### Comparison of the proteomic and metabolomic signatures of S-CAP between the adult and child cohorts

To gain insights into the mechanisms underlying the host responses to S-CAP, we combined data from a published paper [6] to compare the proteomic and metabolomic signatures of S-CAP between the adult and child cohorts. The workflow of data processing and comparison is shown in Additional file 2: Fig. S5. We screened the adult and child S-CAP-specific DEPs and DEMs by comparing S-CAP cases with HCs and NS-CAP cases in the adult and child cohorts, respectively. Then, the overlapping DEPs and DEMs specific to S-CAP in both adults and children were identified for further analysis.

**Fig. 6:**
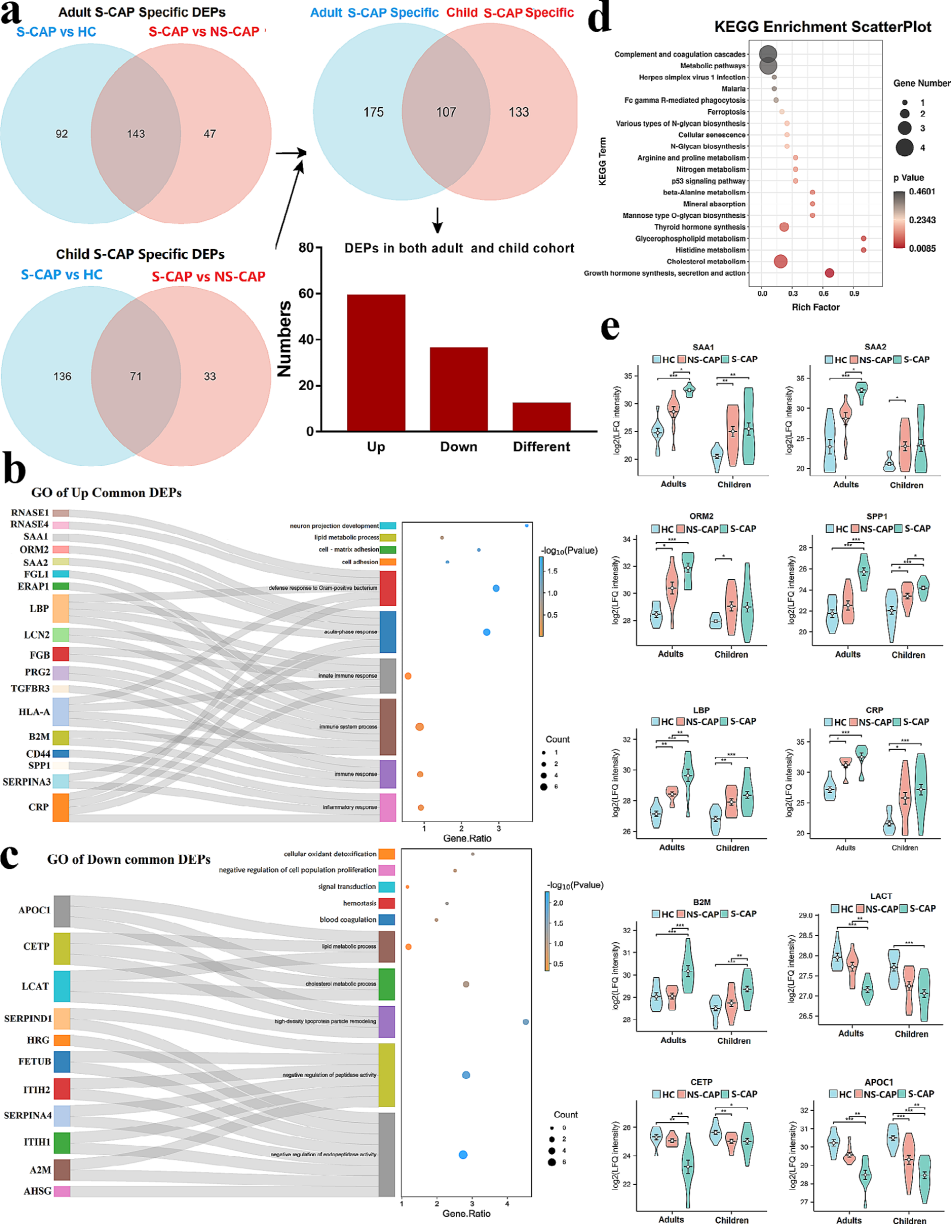
Comparisons of adult and child proteomic data to decipher common features of severe community-acquired pneumonia (S-CAP). (**a**) Venn diagrams showing the number of overlapped differentially expressed proteins (DEPs) in the two datasets and a bar plot showing variation trends. (**b**) Gene ontology (GO) analysis of upregulated common DEPs showing the involved proteins and S-CAP-specific pathways. (**c**) GO analysis of downregulated common DEPs showing the involved proteins and S-CAP-specific pathways. (**d**) Kyoto Encyclopedia of Genes and Genomes (KEGG) analysis of all overlapping DEPs. (**e**) Boxplots showing selected DEPs in the two datasets. Comparisons between two groups were made using Student’s t tests. *P < 0.05, **P < 0.01, and ***P < 0.001

According to our proteomics results, a total of 282 and 240 proteins were differentially expressed in the adult and child S-CAP cohorts, respectively. Among them, 107 DEPs were overlapping (Fig. 6a). As expected, GO analysis indicated that these commonly upregulated DEPs were enriched in the inflammatory response, immune response, and acute-phase response (Fig. 6b). In an opposite manner, downregulated DEPs were mainly involved in metabolism-related pathways, such as lipid metabolic process, cholesterol metabolic process, and high-density lipoprotein particle remodelling (Fig. 6c). Moreover, KEGG pathway analysis also indicated that these common DEPs were enriched in complement and coagulation cascades and metabolic pathways (Fig. 6d). These results indicate that an overactivated inflammatory response is also a key factor involved in the occurrence of severe pneumonia. As shown in Fig. 6e, levels of classical acute-phase proteins, such as ORM2, LBP, and CRP, were clearly elevated in both the adult and child S-CAP cohorts. In addition, SPP1, which is indicative of inflammation and tissue damage [17], was upregulated in S-CAP patients in an age-independent manner. Furthermore, LCAT, CETP, and APOC1, key regulators of lipid metabolic process, were significantly downregulated in both the adult and child S-CAP cohorts. These data confirmed that overactivated inflammation, immune impairment, and metabolic disorders were notable features of S-CAP and contributed to disease severity, irrespective of differences in age.

**Fig. 7:**
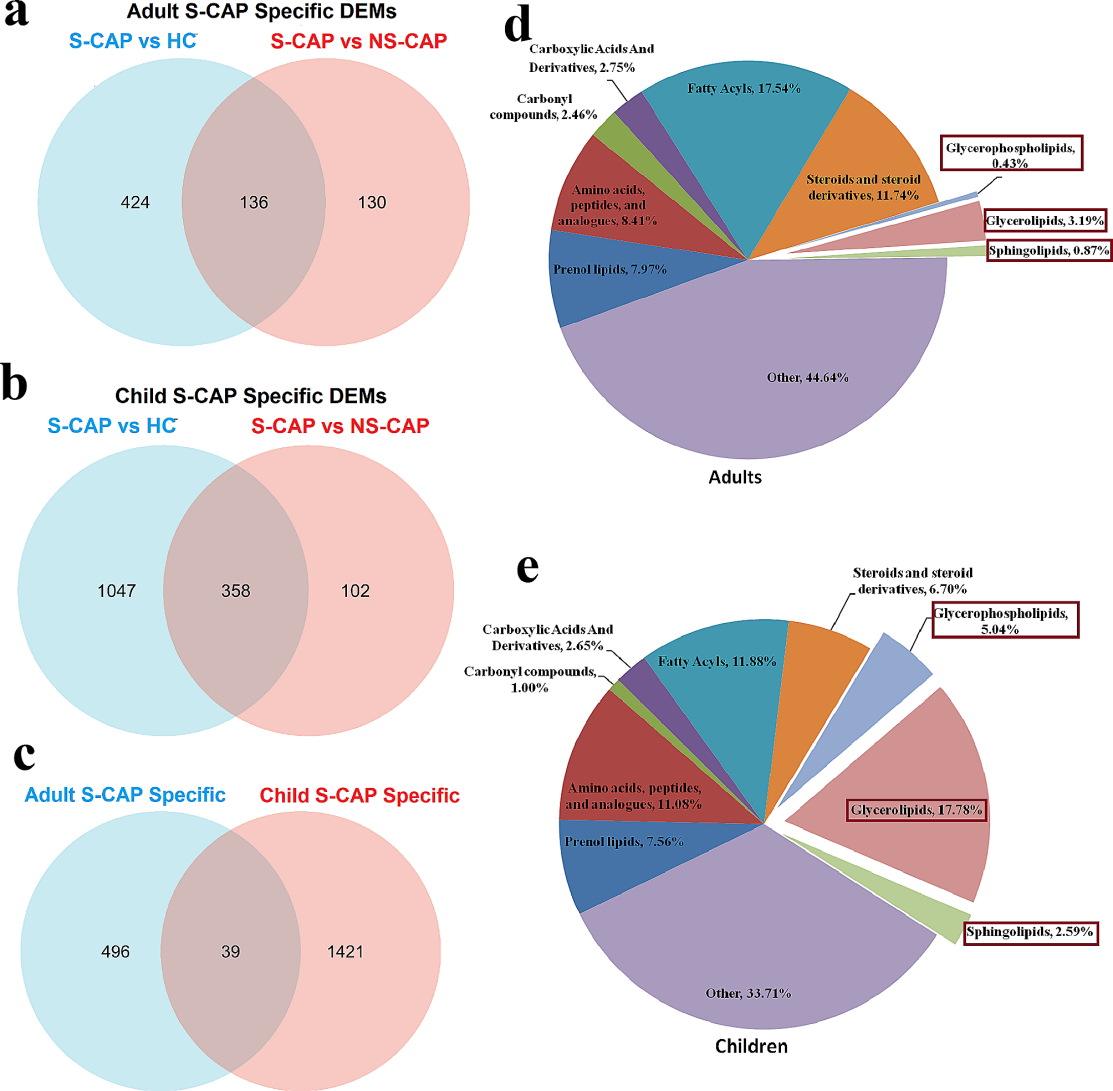
Comparison of adult and child metabolomic data. (**a**) Venn diagram showing the number of adult severe community-acquired pneumonia (S-CAP)-specific differentially expressed metabolites (DEMs). (**b**) Venn diagram showing the number of child S-CAP-specific DEMs. (**c**) Venn diagram showing the overlapping DEMs in the two datasets. (**d**) Pie chart showing the proportion of components of metabolites in adults. (**e**) Pie chart showing the proportion of components of metabolites in children

Additionally, S-CAP-specific DEMs in both the adult and child cohorts were also identified according to the same pattern (Fig. 7a-c). Although there were fewer overlapping DEMs, we interestingly found that the proportion of glycerophospholipids, glycerolipids, and sphingolipids in adult S-CAP cases (Fig. 7d) were lower than that in the child S-CAP cohort (Fig. 7e). Consistently, metabolism is reportedly greatly affected by age and other factors [18, 19]. Overall, although metabolic disorders are involved in the development of severe pneumonia, there are significant differences in metabolic patterns between children and adults.

## Discussion

S-CAP remains a critical public health hazard that is associated with high morbidity and mortality rates [1]. To our knowledge, this study is the first proteomic and metabolomic assessment of S-CAP, as well as the largest blood assessment of S-CAP, in adults to date. In addition to a comprehensive assessment of thousands of proteins that uncovered hundreds of associations with S-CAP progression, these results provide credible evidence for the associations between S-CAP and protein measures. Moreover, these candidate proteins were further validated using an independent validation cohort. Although magnitudes of associations are difficult to directly compare because of the differences in units, the directionality of the associations was consistent for all previously published S-CAP protein biomarkers [20–24]. Moreover, we observed that excessive inflammation was a representative hallmark of adult S-CAP. Inflammation related proteins such as SAA1, SAA2 and CRP were top DEPs in S-CAP pathophysiological processes. We observed that excessive inflammation was a representative hallmark of adult S-CAP. It is reported that two members of which (SAA1 and SAA2) are (along with CRP) the most prominent members of the acute phase response (APR) during which their serum levels rise dramatically after trauma, infection and other stimulation [25]. The Hippo signalling pathway, which was activated in S-CAP patients, has been reported to be associated with acute lung injury [13]. Activated TLR signalling can drive downstream pathways such as NF-κB [26, 27], then lead to the expression of pro-inflammatory cytokines, acute phase proteins, and more [28]. Additionally, the PI3K/Akt signalling pathway plays an important role in multiple diseases and can modulate cellular functions. During viral infection, COVID-19 and Middle East Respiratory Syndrome (MERS) coronavirus could upregulate PI3K/Akt signalling to facilitate viral protein synthesis and promote infection [29, 30]. The PI3K/AKT pathway has also been shown to represent a crucial signalling node during pulmonary fibrosis [31]. Collectively, the hyperactivation of inflammatory pathways in S-CAP is potentially involved in disease exacerbation and late-stage fibrogenesis, making it a promising therapeutic target.

Antibodies derived from plasma cells play a pivotal role in the defence response against severe infection. They can bind to the epitopes of microbes, neutralize toxins, activate complement, and other functions [32]. Here, suppressed acquired immunity was another distinct feature of S-CAP, implying that dysfunctional humoral immunity may contribute to disease progression. This also provides a rationale for intravenous immunoglobulin (IVIG) and passive antibody therapy as an adjunctive treatment method for S-CAP patients.

Protein-metabolite crosstalk analysis was conducted to reveal the potential mechanism of metabolic disorder involvement in severe pneumonia. In this study, we found that S-CAP development is accompanied by significantly downregulated lipid metabolism and upregulated glycolysis. Apolipoproteins are the major components of high-density lipoproteins (HDLs), which can bind and neutralise LPS or lipoteichoic acid [33], inhibit LPS-induced cytokines [34], and downregulate the inflammatory response in macrophages [35]. Critically ill patients have decreased HDL levels, which is related to organ dysfunction [36]. Strikingly, our comparison analysis between the adult and child cohorts further demonstrated that excessive inflammation and dysregulated lipid metabolism are common features, independent of age or population. Furthermore, elevated lactate levels are significantly associated with disease severity [37]. Lactate may exert immunosuppressive effects [38], such as promoting macrophage polarisation to the M2 phenotype. Lactate production during glycolysis can also contribute to histone lactylation and epigenetic modifications that stimulate gene transcription and further influence immune cell functions [39]. Thus, reduced HDL/lipid metabolism and elevated glycolysis can lead to excessive inflammation and dysregulated defence responses, which may be promising biomarkers for monitoring and therapeutically targeting S-CAP. Moreover, levels of (4R,6 S)-6-[(E)-2-[2-(4-Fluoro-3-methylphenyl)-4,6-dimethylphenyl]ethenyl]-4-hydroxyoxan-2-one, doxofylline and armillane were the obviously changed in S-CAPs compared to controls. Doxofylline is a newer generation xanthine with both bronchodilating and anti-inflammatory activities [40]. Here, elevated doxofylline might represent the anti-inflammatory activities were stimulated. There are limited reports on the function of the other two metabolites.

It should be noted that the sample types in the adult and child cohorts were different. The samples in the adult cohort were plasma, while those in the child cohort were serum. However, it has been shown that the differences of proteomic data are mainly from coagulation [41], and 93.5% of compounds also overlapped in the metabolic data [42] between serum and plasma samples. Furthermore, this was a single-centre prospective study. Future larger cohort studies using tests with higher sensitivity are warranted to confirm the findings of our work here.

## Conclusion

In this study, we provided a comprehensive multi-omics investigation of these patients. Our results suggest that excessive inflammation, suppressed humoral immunity, and disordered lipid metabolism are involved in the development of severe pneumonia. Our data deepen the overall understanding of the specific molecular alterations and pathogenesis that underly S-CAP, which will be helpful for developing future therapies.

### Electronic supplementary material

Below is the link to the electronic supplementary material.

**Supplementary Material 1: Table S1.1.** Additional characteristics of NS-CAP patients, Related to Figure 1. **Table S1.2.** Additional characteristics of S-CAP patients, Related to Figure 1. **Table S1.3.** Additional characteristics of DCs, Related to Figure 1. **Table S1.4.** Additional characteristics of HCs, Related to Figure 1

**Supplementary Material 2: Fig. S1.** Summary the sample collection timing of NS-CAP patients (n=43) and S-CAP patients (n=31). The y-axis displays patient identification numbers; the x-axis shows days since disease onset. **Fig. S2.** Detected proteins in different samples. Distribution of the number of a) quantified proteins and b) peptides in the 40 plasma samples from the training cohort. **Fig. S3.** Quality control of proteomic data in the training cohort. a) Score plot and parameters of the partial least squares-discriminate analysis (PLS-DA) model for severe community-acquired pneumonia (S-CAP) cases and healthy controls (HCs). b) Score plot and parameters of the PLS-DA model for S-CAP cases and disease controls (DCs). c) Score plot and parameters of the PLS-DA model for S-CAP cases and non-severe (NS)-CAP cases. **Fig. S4.** Quality control of metabolomic data. a) Score plot and parameters of the partial least squares-discriminate analysis (PLS-DA) model for severe community-acquired pneumonia (S-CAP) cases and healthy controls (HCs). b) Score plot and parameters of the PLS-DA model for S-CAP cases and disease controls (DCs). c) Score plot and parameters of the PLS-DA model for S-CAP cases and non-severe (NS)-CAP cases. **Fig. S5.** Workflow for determining common differentially expressed proteins (DEPs) or differentially expressed metabolites (DEMs) from adult and child severe community-acquired pneumonia (S-CAP) patients. We screened adult and child S-CAP-specific DEPs and DEMs by comparing S-CAP cases with healthy controls (HCs) and non-severe (NS)-CAP cases in the adult and child cohorts, respectively. Then, the overlapping DEPs and DEMs specific to S-CAPs in both the adult and child cohorts were identified for further analysis

**Supplementary Material 3: Table S2.** The clinical information and conducted biochemical laboratory tests

**Supplementary Material 4: Table S3.1.** Differentially expressed proteins. **Table S3.2.** Differentially expressed metabolites

## Data Availability

Correspondence and requests for data should be addressed to Prof. Jieqiong Li and Prof. Zhaohui Tong.

## Abbreviations

APOA1: Apolipoprotein A 1
AUC: Area under the curve
CAP: Community-acquired pneumonia
CETP: Cholesteryl ester transfer protein
CRP: C-reactive protein
DCs: Diseases controls
DEMs: Differentially expressed metabolites
DEPs: Differentially expressed proteins
ELISA: Enzyme-linked immunosorbent assay
GO: Gene Ontology
GPI: Glucose-6-phosphate isomerase
HCs: Healthy controls
HDLs: High-density lipoproteins
ICU: Intensive care unit
IGHG1: Immunoglobulin heavy constant gamma 1
ITIH1: Inter-alpha-trypsin inhibitor heavy chain H1
IVIG: Intravenous immunoglobulin
KEGG: Kyoto Encyclopedia of Genes and Genomes
LBP: Lipopolysaccharide-binding protein
LCAT: Phosphatidylcholine-sterol acyltransferase
LDHA: L-lactate dehydrogenase A
MERS: Middle East Respiratory Syndrome
NS-CAP: Non-severe community-acquired pneumonia
ORM1: Alpha-1-acid glycoprotein
PCA: Principal component analysis
PI3K-Akt: Phosphatidylinositol 3-kinase/ serine-threonine kinase
PLS-DA: Partial least squares-discriminate analysis
S-CAP: Severe community-acquired pneumonia
SAA1: Serum amyloid A-1 protein
SFTPB: Surfactant-associated protein B
TKT: Transketolase
TLR: Toll-like receptor
