# Supplementary material for: Multi-omics analysis reveals overactive inflammation and dysregulated metabolism in severe community-acquired pneumonia patients
Source: Respir Res. 2024 Jan 19;25:45. doi: 10.1186/s12931-024-02669-6 (PMC10797892; doi:10.1186/s12931-024-02669-6)
Supplement: Supplementary file 2 — Supplementary Material 2: Fig. S1. Summary the sample collection timing of NS-CAP patients (n=43) and S-CAP patients (n=31). The y-axis displays patient identification numbers; the x-axis shows days since disease onset. Fig. S2. Detected proteins in different samples. Distribution of the number of a) quantified proteins and b) peptides in the 40 plasma samples from the training cohort. Fig. S3. Quality control of proteomic data in the training cohort. a) Score plot and parameters of the partial least squares-discriminate analysis (PLS-DA) model for severe community-acquired pneumonia (S-CAP) cases and healthy controls (HCs). b) Score plot and parameters of the PLS-DA model for S-CAP cases and disease controls (DCs). c) Score plot and parameters of the PLS-DA model for S-CAP cases and non-severe (NS)-CAP cases. Fig. S4. Quality control of metabolomic data. a) Score plot and parameters of the partial least squares-discriminate analysis (PLS-DA) model for severe community-acquired pneumonia (S-CAP) cases and healthy controls (HCs). b) Score plot and parameters of the PLS-DA model for S-CAP cases and disease controls (DCs). c) Score plot and parameters of the PLS-DA model for S-CAP cases and non-severe (NS)-CAP cases. Fig. S5. Workflow for determining common differentially expressed proteins (DEPs) or differentially expressed metabolites (DEMs) from adult and child severe community-acquired pneumonia (S-CAP) patients. We screened adult and child S-CAP-specific DEPs and DEMs by comparing S-CAP cases with healthy controls (HCs) and non-severe (NS)-CAP cases in the adult and child cohorts, respectively. Then, the overlapping DEPs and DEMs specific to S-CAPs in both the adult and child cohorts were identified for further analysis [file 12931_2024_2669_MOESM2_ESM.docx]

**Supplemental Figures**

**Multi-omics Analysis Reveals Overactive Inflammation and Dysregulated Metabolism in Severe Community-Acquired Pneumonia Patients**

Jieqiong Li**^1,^** **^†, *^**, Yawen Wang**^2, 3, †^**, Weichao Zhao**^2, 4,^ ^†^**, Tingyu Yang**^1^**, Qianyu Zhang**^2^**, Huqin Yang**^2^**, Xuyan Li**^2^**, Zhaohui Tong**^2,^ ^*^**

^1^ Medical Research Center, Beijing Institute of Respiratory Medicine and Beijing Chao-Yang Hospital, Capital Medical University, Beijing, China.

^2^ Department of Respiratory and Critical Care Medicine, Beijing Institute of Respiratory Medicine and Beijing Chao-Yang Hospital, Capital Medical University, Beijing, China.

^3^ Department of Respiratory and Critical Care Medicine, Tianjin Chest Hospital, Tianjin, China.

^4^ Department of Respiratory Medicine, Strategic Support Force Medical Center, Beijing, China.

**^†^** Contributed equally to this manuscript.

**^*^** Corresponding author:

Zhaohui Tong, Email: tongzhaohuicy@sina.com

Jieqiong Li, Email: jieqiongli2010@163.com

8 Workers Stadium South Road, Chaoyang District, Beijing

**Fig. S1**


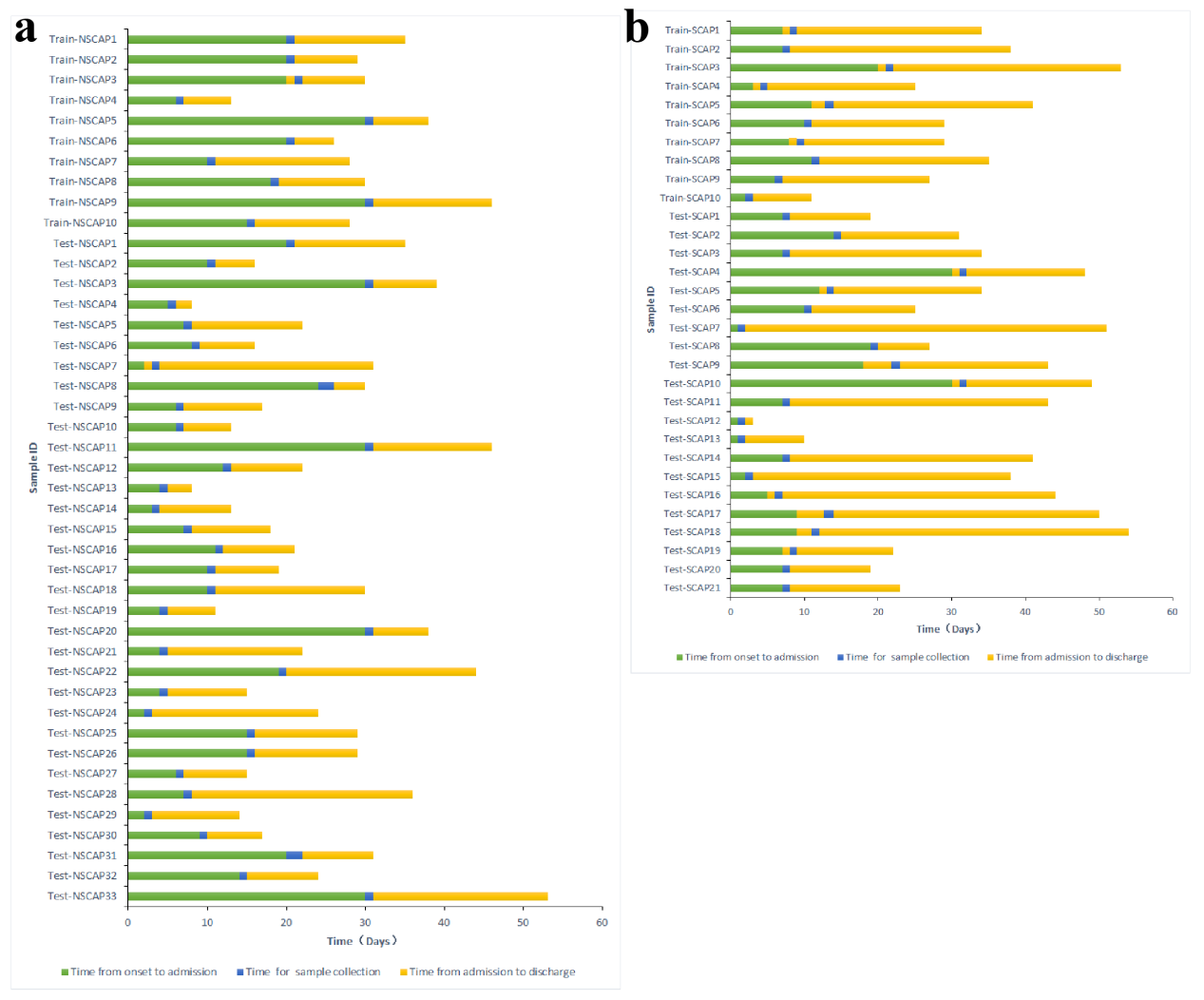


**Fig. S1**. Summary the sample collection timing of NS-CAP patients (n=43) and S-CAP patients (n=31). The y-axis displays patient identification numbers; the x-axis shows days since disease onset.

**Fig. S2**

**
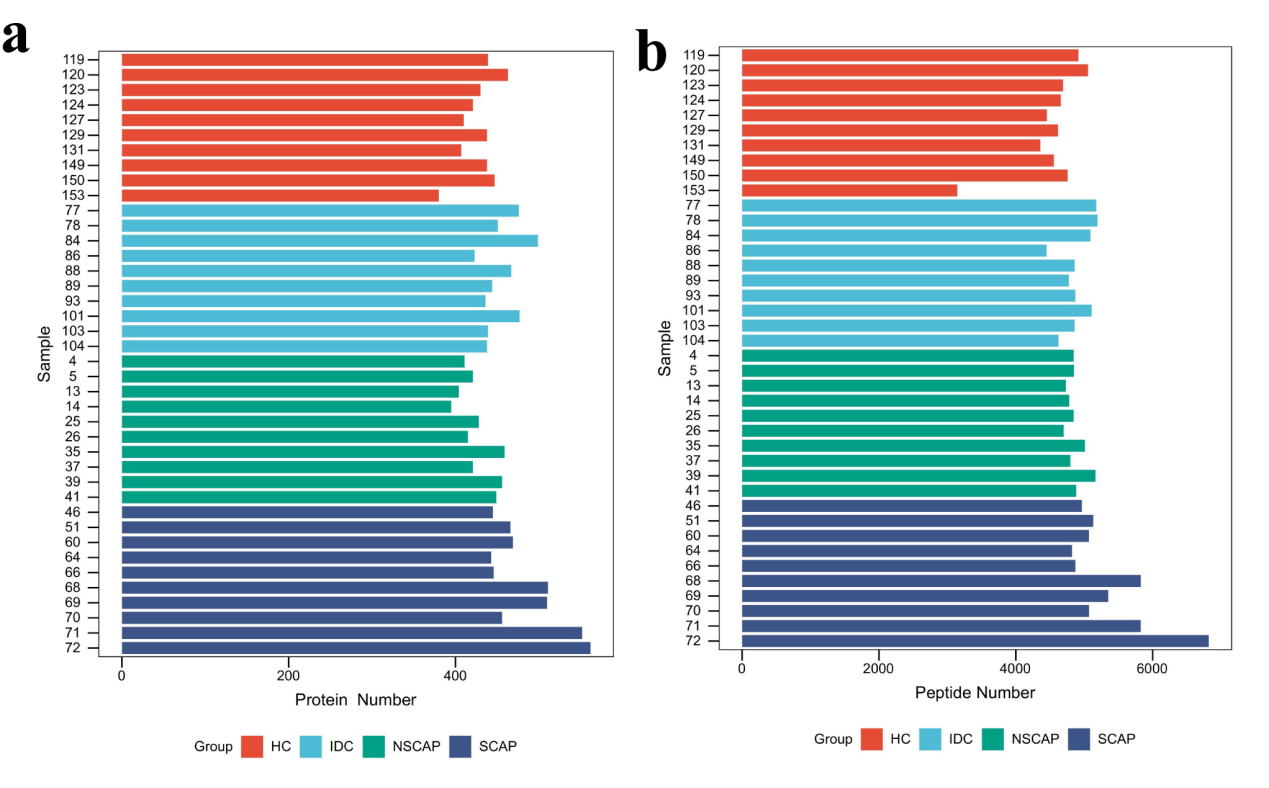
**

**Fig. S2.** Detected proteins in different samples. Distribution of the number of a) quantified proteins and b) peptides in the 40 plasma samples from the training cohort.

**Fig. S3**


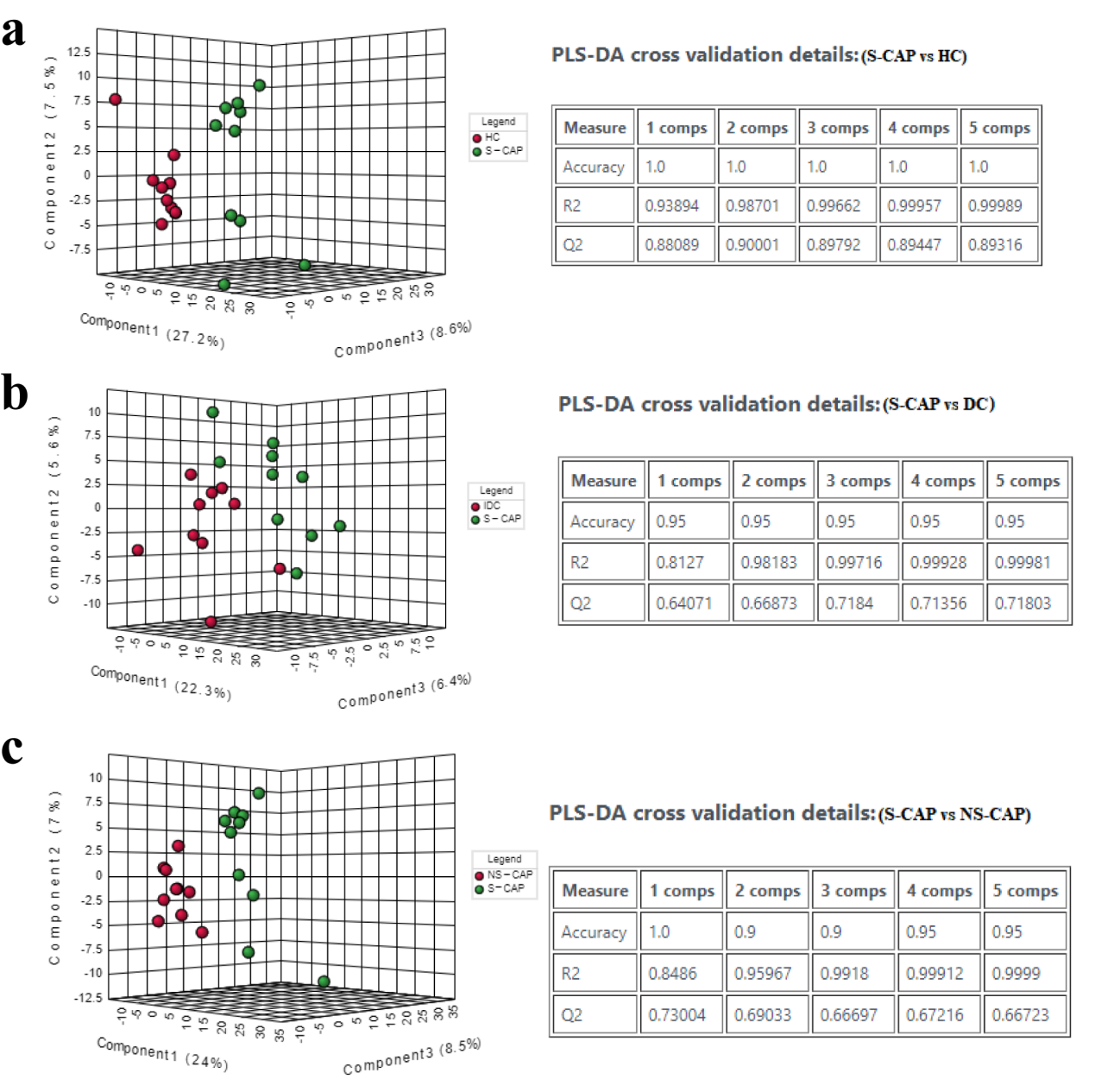


**Fig. S3.** Quality control of proteomic data in the training cohort. a) Score plot and parameters of the partial least squares-discriminate analysis (PLS-DA) model for severe community-acquired pneumonia (S-CAP) cases and healthy controls (HCs). b) Score plot and parameters of the PLS-DA model for S-CAP cases and disease controls (DCs). c) Score plot and parameters of the PLS-DA model for S-CAP cases and non-severe (NS)-CAP cases.

**Fig. S4**


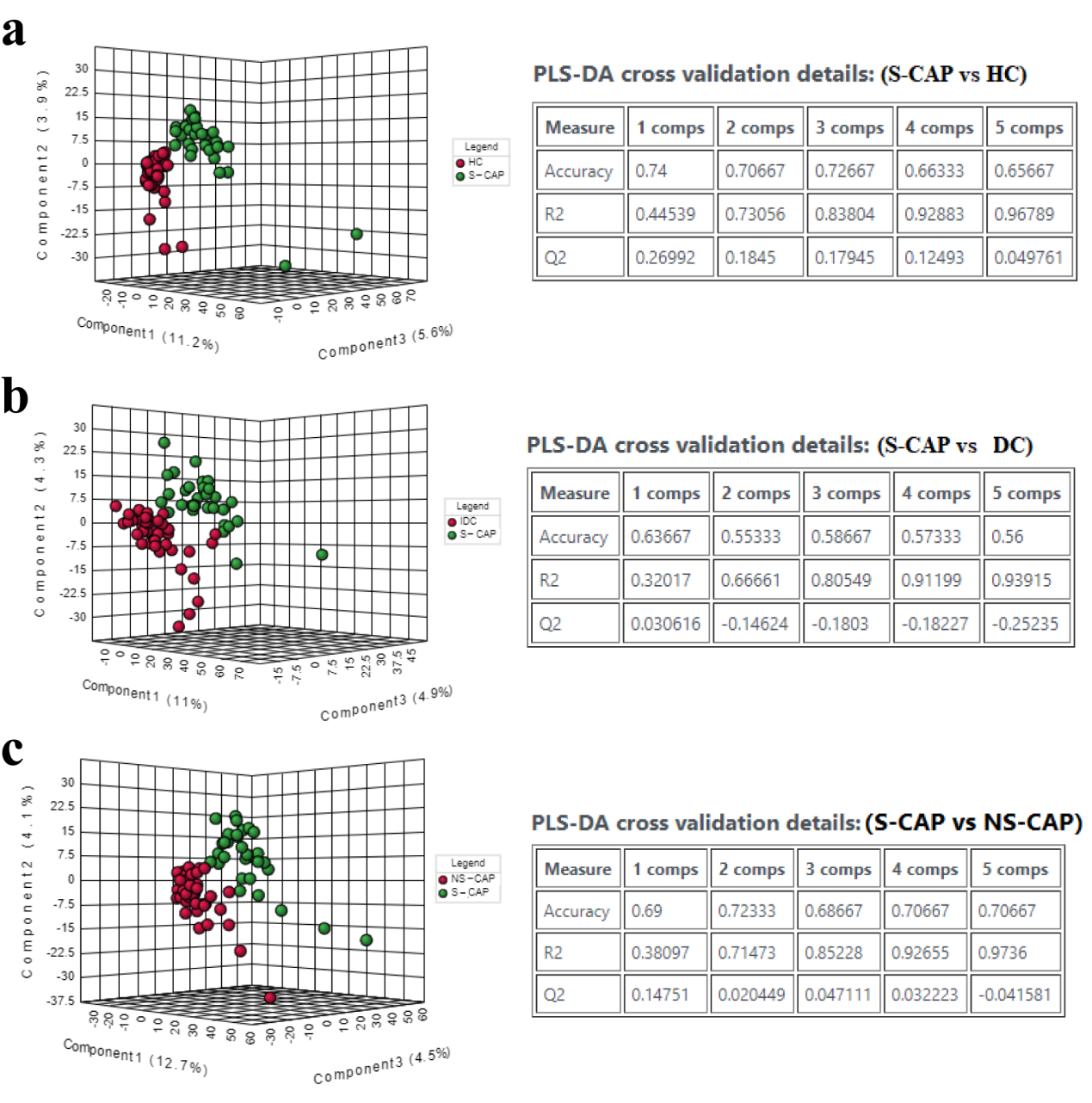


**Fig. S4.** Quality control of metabolomic data. a) Score plot and parameters of the partial least squares-discriminate analysis (PLS-DA) model for severe community-acquired pneumonia (S-CAP) cases and healthy controls (HCs). b) Score plot and parameters of the PLS-DA model for S-CAP cases and disease controls (DCs). c) Score plot and parameters of the PLS-DA model for S-CAP cases and non-severe (NS)-CAP cases.

**Fig. S5**


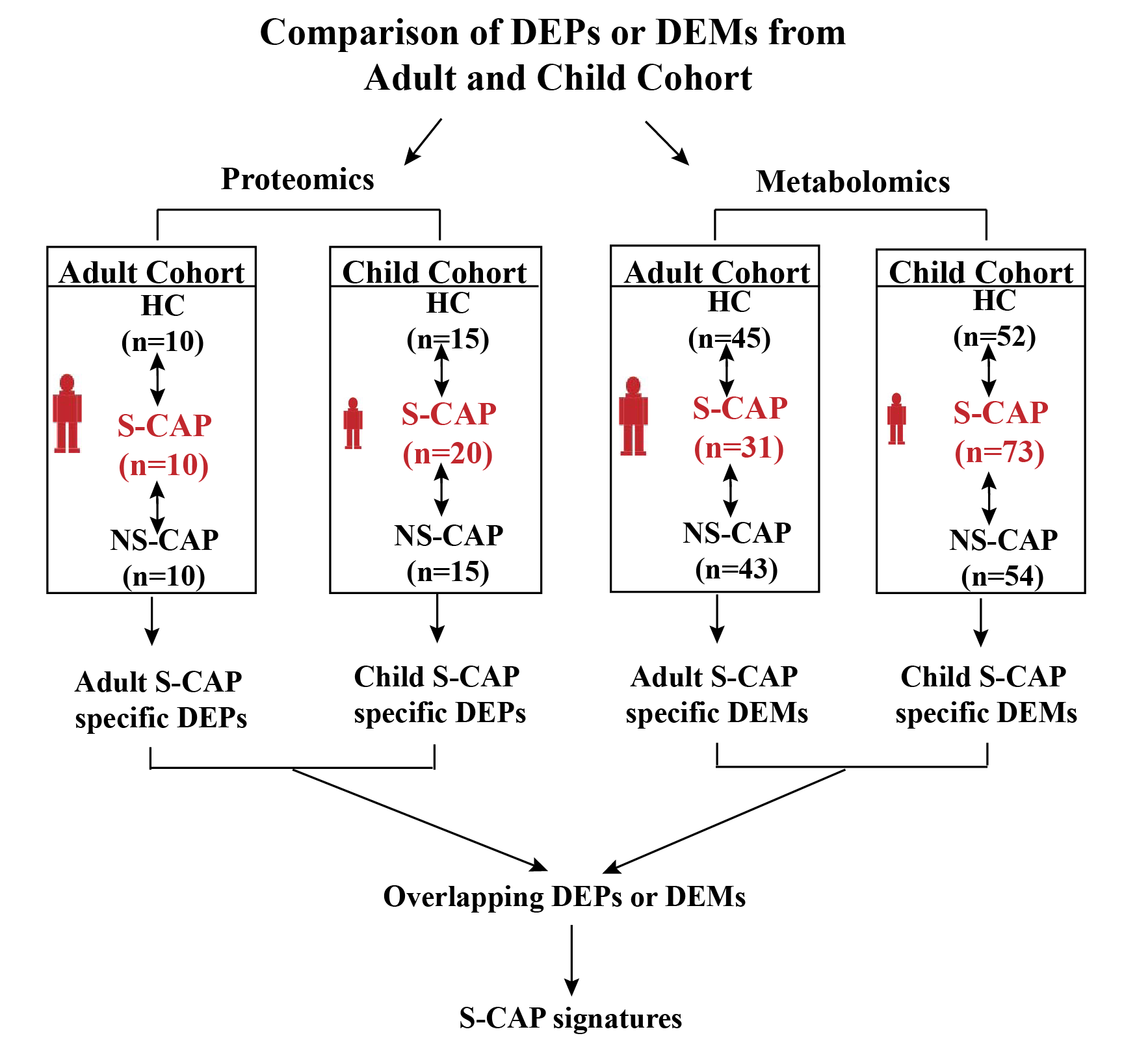


**Fig. S5.** Workflow for determining common differentially expressed proteins (DEPs) or differentially expressed metabolites (DEMs) from adult and child severe community-acquired pneumonia (S-CAP) patients. We screened adult and child S-CAP-specific DEPs and DEMs by comparing S-CAP cases with healthy controls (HCs) and non-severe (NS)-CAP cases in the adult and child cohorts, respectively. Then, the overlapping DEPs and DEMs specific to S-CAPs in both the adult and child cohorts were identified for further analysis.
