# Supplementary material for: Multi-omics analysis reveals overactive inflammation and dysregulated metabolism in severe community-acquired pneumonia patients
Source: Respir Res. 2024 Jan 19;25:45. doi: 10.1186/s12931-024-02669-6 (PMC10797892; doi:10.1186/s12931-024-02669-6)
Supplement: Supplementary file 3 — Supplementary Material 3: Table S2. The clinical information and conducted biochemical laboratory tests [file 12931_2024_2669_MOESM3_ESM.docx]

Table S2: The clinical information and conducted biochemical laboratory tests.

| Characteristics | Training Cohort (n=40) | | | | | Testing Cohort (n=121) | | | | |
| --- | --- | --- | --- | --- | --- | --- | --- | --- | --- | --- |
|  | S-CAP  (n=10) | NS-CAP  (n=10) | IDC  (n=10) | CON  (n=10) | P value a | S-CAP  (n=21) | NS-CAP  (n=33) | IDC  (n=32) | CON  (n=35) | P value a |
| Gender (male/female) | 7/3 | 7/3 | 7/3 | 7/3 | 1.000 | 17/4 | 23/10 | 21/11 | 19/16 | 0.486 |
| Age (years) | 68.5±10.4 | 54.9±11.3 | 63.9+10.4 | 63.3±11.0 | 0.027 | 61.5±15.9 | 56.3±17.8 | 64.8±11.4 | 57.1±9.3 | 0.087 |
| Days of hospitalization | 22.1±6.7 | 10.3±4.1 | 16.4±7.1 | / | 0.001 | 23.7±14.3 | 10.6±6.1 | 11.5±7.41 | / | <0.001 |
| ICU admission, n (%) | 10 (100) | 0 (0) | 1 (10) | / | <0.001 | 21 (100) | 0(0) | 2 (6.25) | / | <0.001 |
| Invasive ventilation, n (%) | 9 (90) | 0 (0) | 1 (10) | / | <0.001 | 14 (66.67) | 0(0) | 1 (3.03) | / | <0.001 |
| High flow oxygen uptake, n (%) | 8 (80) | 0 (0) | 1 (10) | / | <0.001 | 15 (71.4) | 3 (9.1) | 3 (9.4%) | / | <0.001 |
| Inflammation markers b | | | | | | | | | | |
| PCT, ng/mL | 5.9±13.6 | 0.06±0.03 | 1.5±2.7 | / | 0.294 | 9.9±20 | 0.56±2.25 | 0.05±0.04 | / | 0.010 |
| CRP,mg/L | 123.8±70.5 | 49.1±62.3 | 54.6±66.0 | / | 0.045 | 133.9±105.9 | 53.8±80.1 | 17.4±35.0 | / | <0.001 |
| WBC, 10^9^/L | 12.4±5.6 | 6.7±2.2 | 8.1±2.5 | / | 0.005 | 11.6±7.3 | 7.2±3.6 | 6.0±2.8 | / | <0.001 |
| Lym, % | 6.0±3.52 | 27.3±9.6 | 17.62±7.3 | / | <0.001 | 8.6±6.2 | 22.8±12.5 | 24.1±11.0 | / | <0.001 |
| Neu, % | 90.1±7.8 | 62.35±11.4 | 72.56±8.4 | / | <0.001 | 86.3±9.4 | 66.6±14.7 | 63.1±16.1 | / | <0.001 |
| PH | 7.4+0.1 | 7.4±0.02 | 7.4±0.03 | / | 0.794 | 7.1±1.6 | 7.2±1.3 | 5.1±3.5 | / | 0.001 |
| CO_2_, mmHg | 31.4±6.5 | 39.1±5.5 | 76.1±13.7 | / | <0.001 | 35.0±14.8 | 35.2±8.4 | 57.2±42.0 | / | 0.002 |
| O_2_, mmHg | 81.9±26.3 | 91.5±24.1 | 37.9±6.6 | / | <0.001 | 72.9±28.8 | 81.4±28.2 | 29.2±22.4 | / | 0.000 |
| FiO2 | 0.5±0.2 | 0.2±0.04 | 0.2±0.0 | / | <0.001 | 0.5±0.3 | 0.3±0.1 | 0.2±0.1 | / | 0.000 |

^a^ P value among these groups; ^b^ Data are presented as mean ± SD.

PCT, procalcitonin; CRP, C-reaction protein; WBC, white blood cell; Neu %, neutrophil %; Lym %, lymphocyte%. DC, disease control; HC, healthy control; S-CAP; severe community-acquired pneumonia. NS-CAP, non-severe community-acquired pneumonia.
